# First annual congress of the Faculty of Consulting Physicians of South Africa, 18–20 May 2012

**Published:** 2012-07

**Authors:** Naomi Rapeport

**Affiliations:** Milpark Hospital, Johannesburg, South Africa

The specialist physician has always played a pivotal role in the healthcare of South Africans. Following specialisation, either through the College of Medicine or a Master’s of Medicine obtained from a university, many of these doctors have branched out into the private sector and continue to play a vital role in the management of patients.

The total number of specialist physicians registered with the Health Professions Council of South Africa (HPCSA) in both the public and private sector is fewer than 600 and these doctors provide specialist healthcare for a population of over 40 million people. Over the past four decades there has been a major ‘brain drain’ of academic and private physicians to North America, Australia and New Zealand and this resultant shortage of specialists places an enormous burden on those who have remained in South Africa.

Due to the burgeoning epidemic of HIV and AIDS in our country, patients with AIDS-associated conditions have swamped medical admission wards, placing a huge load on clinicians. The increase in prevalence of non-communicable diseases such as diabetes mellitus and coronary artery disease in our previously disadvantage populations has also contributed to an increased work load as our population undergoes epidemiological transition from famine and pestilence to degenerative and man-made disease. South Africa now has the distinction of having one of the most obese populations in the world.

All private-practice doctors are required to be registered with the HPCSA as well as the Board of Healthcare Funders (BHF), who are responsible for issuing MP and private practice numbers, respectively. The latter is known as the practice code numbering system (PCNS). The HPCSA, a statutory body, established in terms of the Health Professions Act No. 56, is committed to serving and protecting the public. It regulates the health profession in aspects pertaining to registration, education and training, professional conduct and ethical behaviour, continuing professional development, and fostering compliance with healthcare standards. Neither of these organisations is involved in, or has the interests of the private practitioner at heart. This is the role of the various professional associations.

Previously, as far as consulting physicians were concerned, matters relating to cost coding for medical services, procedures and the introduction of new codes were the responsibility of the private practice committee of the South African Medical Association (SAMA) and in particular the Association of Physicians. Due to the political milieu of South Africa prior to the abolition of apartheid, the Medical Association did not represent the interest of most of our fellow colleagues and in particular, the Association of Physicians did not have any active membership. It consisted of a lone physician who did all the coding work.

In 1997, a group of concerned specialist physicians in Johannesburg, under the leadership of Dr Naomi Rapeport, established the Faculty of Consulting Physicians of South Africa (FCPSA) to try and unite all specialist physicians in private practice into one professional group. No accurate data were available from the HPCSA, SAMA or BHF as to how many physicians were practicing in South Africa. Through networking with colleagues in all the different provinces, a nucleus of physicians was established. The work of coding, and interaction with SAMA, BHF and HPCSA was undertaken by the Faculty, who took over the role of the defunct Association of Physicians.

Countless hours were expended on the workings of the organisation. Annual academic meetings were held from 1998 to 2002 in the major centres, and regular updates were sent to members. Although there was a constant drive to encourage doctors to join the Faculty, it was a persistent uphill battle. To date, many doctors in private practice have no idea what an essential role the organisation plays in terms of their daily practice management.

In 2004, Dr Adri Kok took over the leadership of the organisation from Dr Rapeport. The faculty has expanded to include dermatologists, rheumatologists, pulmonologists, neurologists and nephrologists in private practice, and consulting physicians in the public sector who do limited private practice. Through tireless ongoing effort, Dr Kok has continued to head and run the organisation.

Due to major changes in the private practice arena, ranging from the introduction of prescribed minimum benefits (PMB), formation of the Council of Medical Schemes (CMS), the ruling of the Compensation Commissioner on the so-called collusion of doctors’ fees, and conflict between the Department of Health and SAMA regarding ownership of coding, the Faculty has continued to play a vital role. The organisation, together with many of the other medical and surgical disciplines, have left SAMA and now operate under the umbrella of the South African Private Practitioners Forum (SAPPF) and continue to represent the private practice specialist.

This umbrella group together with private hospital groupings was awarded costs in a court case against the Department of Health regarding ownership of coding in 2010. The case was to challenge the validity of the reference price list (RPL) in its present form. Despite numerous meetings with assigned colleagues of the Department of Health to resolve this issue, it ended in a stalemate.

The RPL is a list of fees for medical professionals based on service and procedure codes, as detailed in the SAMA Doctors’ Guide to Billing. The Department of Health tried to take over ownership of the RPL without having any experience of the workings of the coding system. They did not take into account issues such as the practice costs incurred by doctors (many practices have huge practice overheads), which was validated by a practice cost study submission undertaken by the professional groupings.

Medical schemes also choose their rates of reimbursement on a basis that they consider to be reasonable, based on the RPL. It directly affects the amount which privately insured patients will be able to recoup from their medical schemes for the cost of health services. New codes are introduced as clinical practice expands and changes.

The SAPPF with SAMA members have assessed approximately 600 codes for validation. The hope of the future is the compilation of a new South African procedure coding structure which will accurately describe consultations and procedures. Fees charged by service providers are governed by the Health Professions Act of 1974. However there is also the issue of the Competition Act of 1998, which enforces anti-competitive behaviour by the profession, and this states that doctors may not conduct unfair, collusive and undesirable business practices by all charging the same fees.

The organisation has been involved in designing updated algorithms for PMB conditions. This is the minimum set of healthcare benefits to which all patients contributing to medical schemes are entitled. Meetings with the CMS have been held and evidence-based medicine outcomes shown to motivate appropriate revisions of the PMB algorithms. As practicing clinicians, best practice is encouraged and doctors from the different groups have been involved in updating clinical guidelines. This includes the lipid and diabetes guidelines, to name a few. The organisation has been involved in reviewing the green paper of the National Health Insurance. It also has joined the International Society of Internal Medicine, which was originally established in 2004, and is one of three country members from Africa.

The first annual congress of the FCPSA was held in Cape Town recently. It was very well received by all attending colleagues and the calibre of lectures was excellent. Local speakers from the private sector and academic institutions, as well as international speakers covered a wide range of subjects pertinent to practicing physicians. A number of training medical registrars from different universities were sponsored to attend the meeting and they were surprised by the extremely high academic standard.

